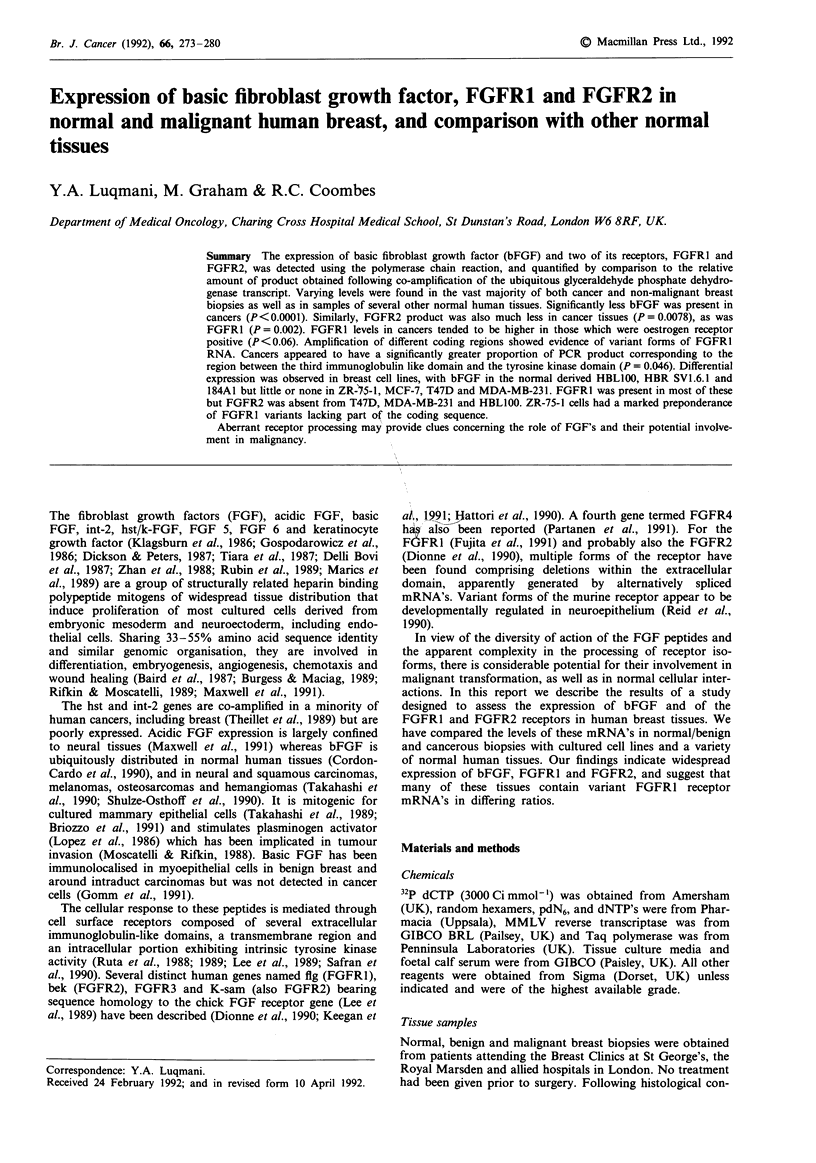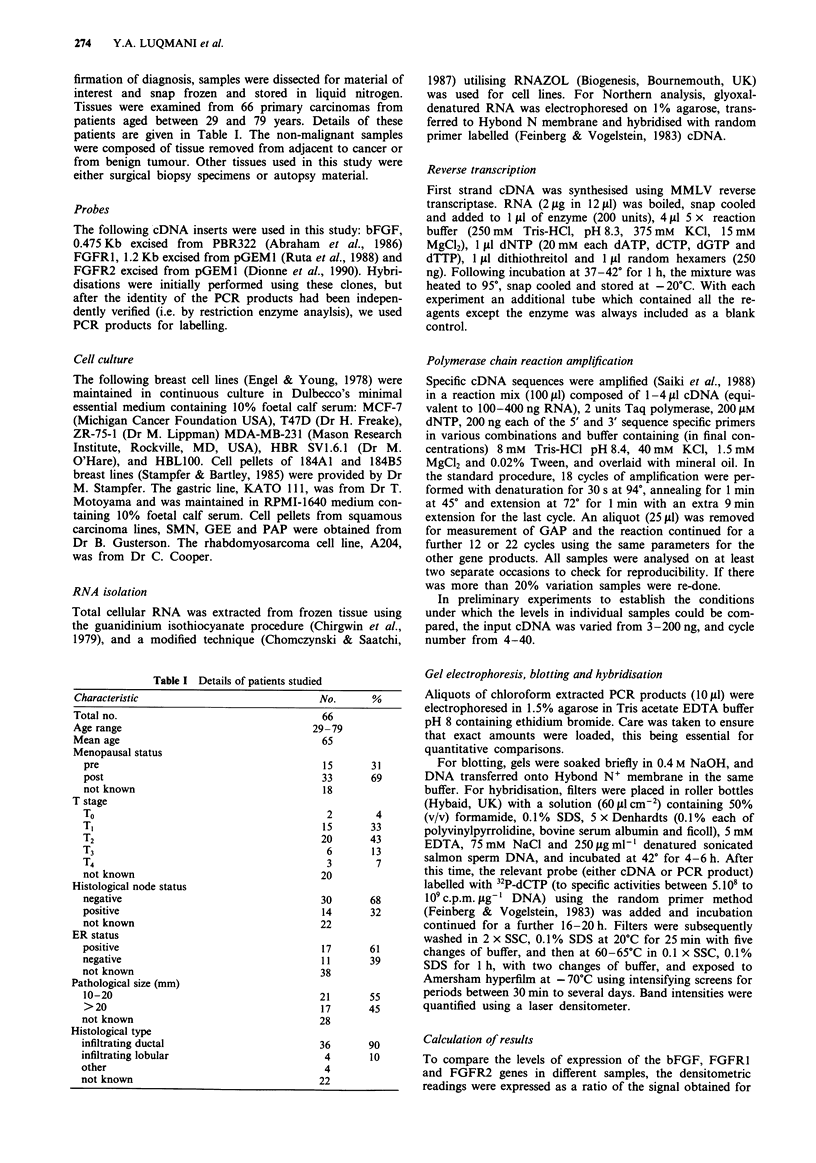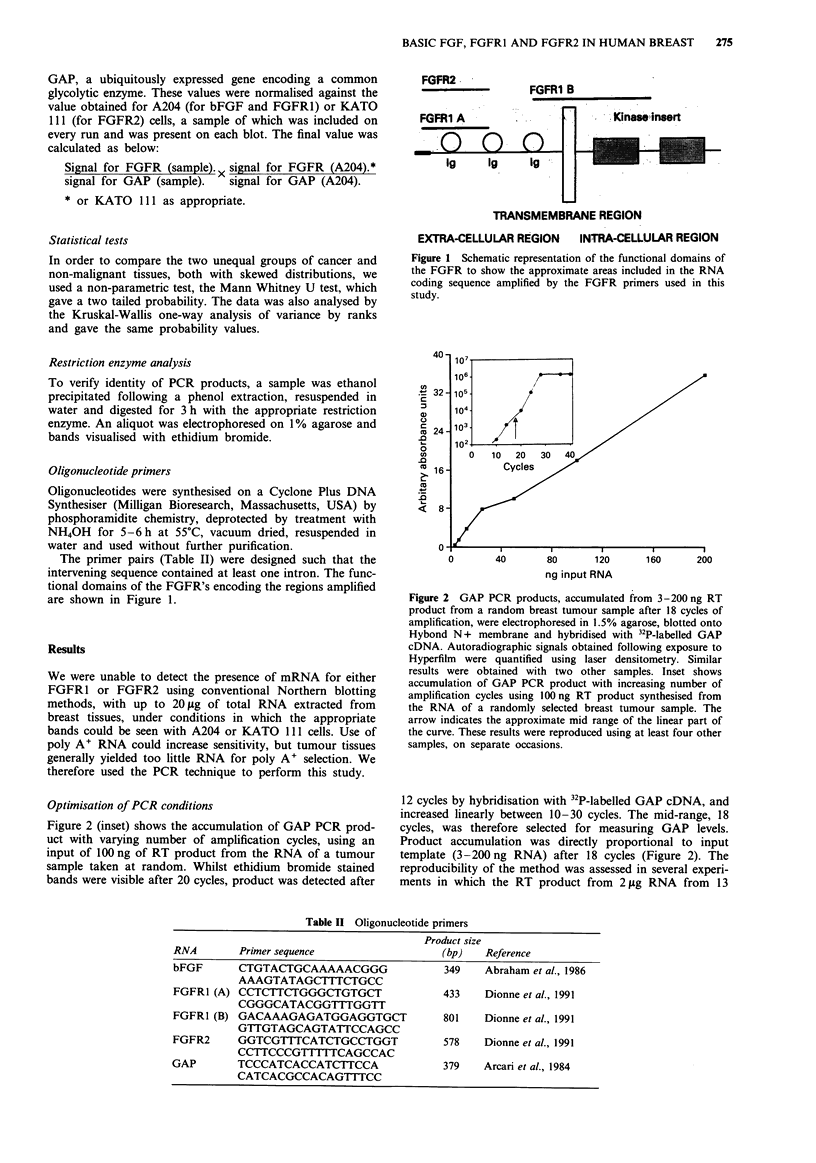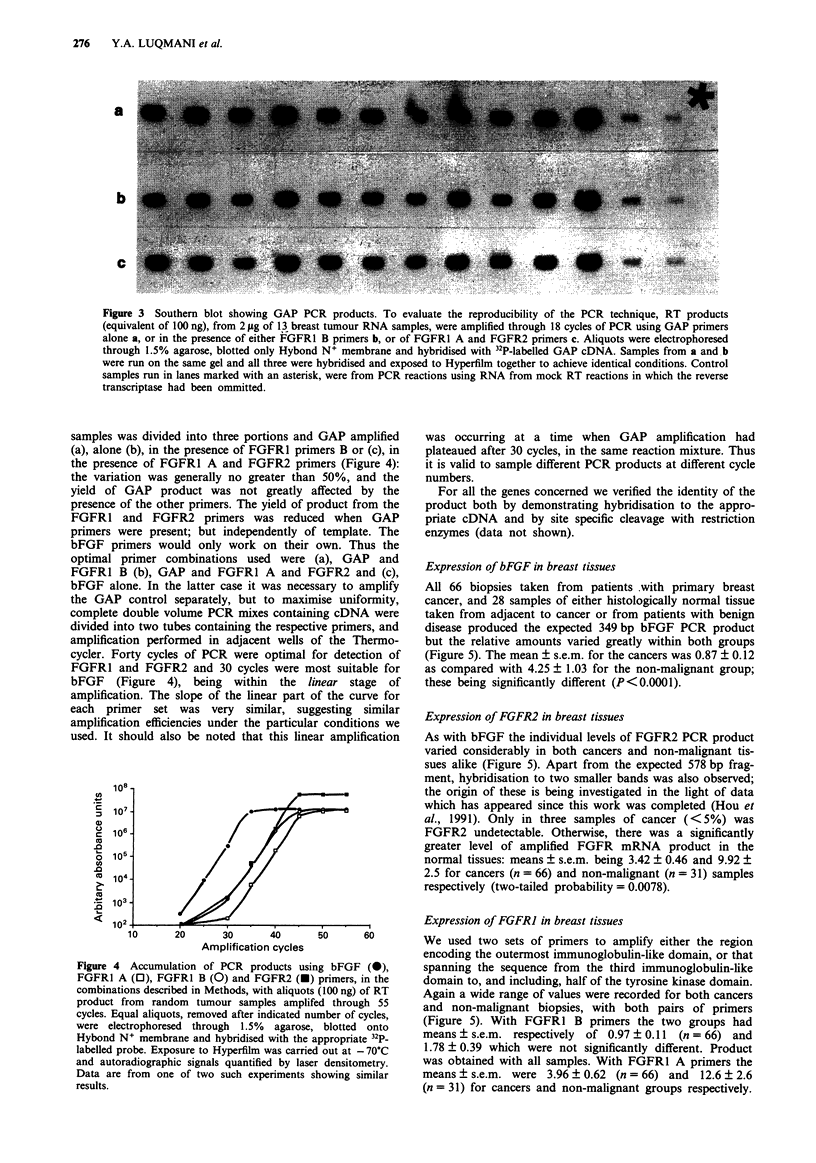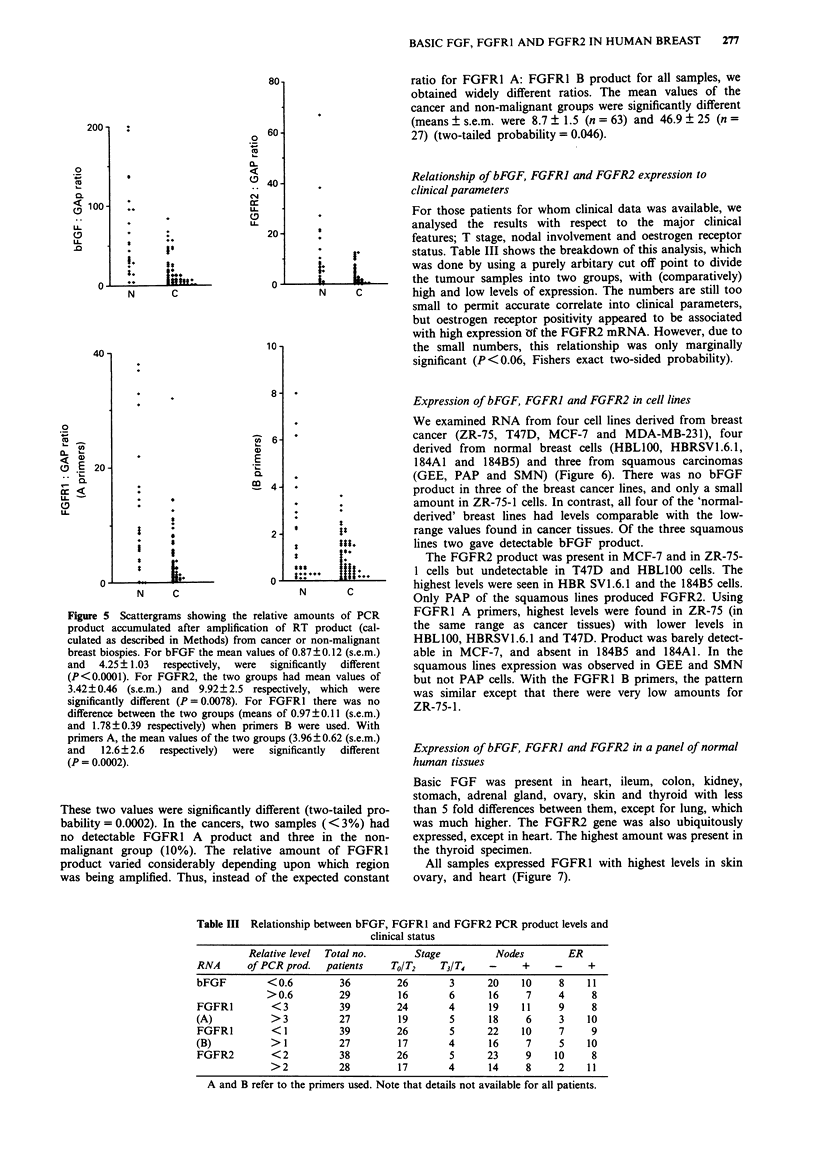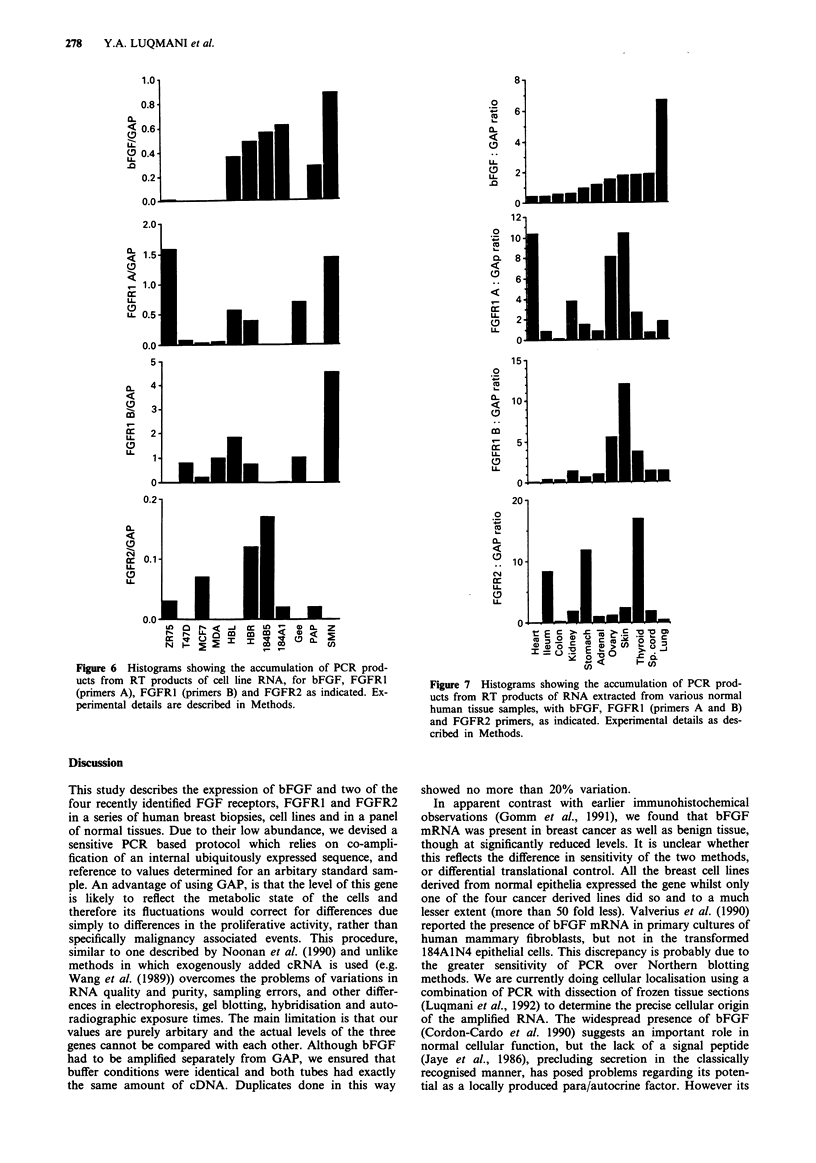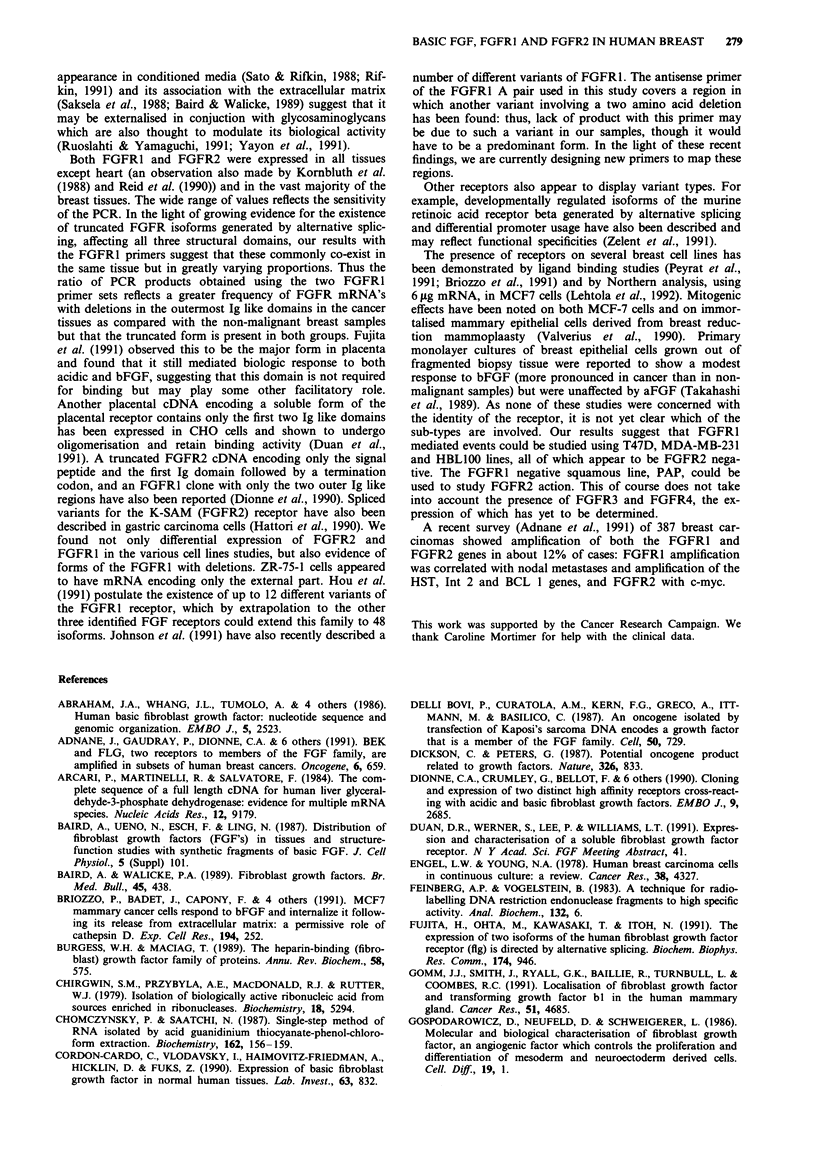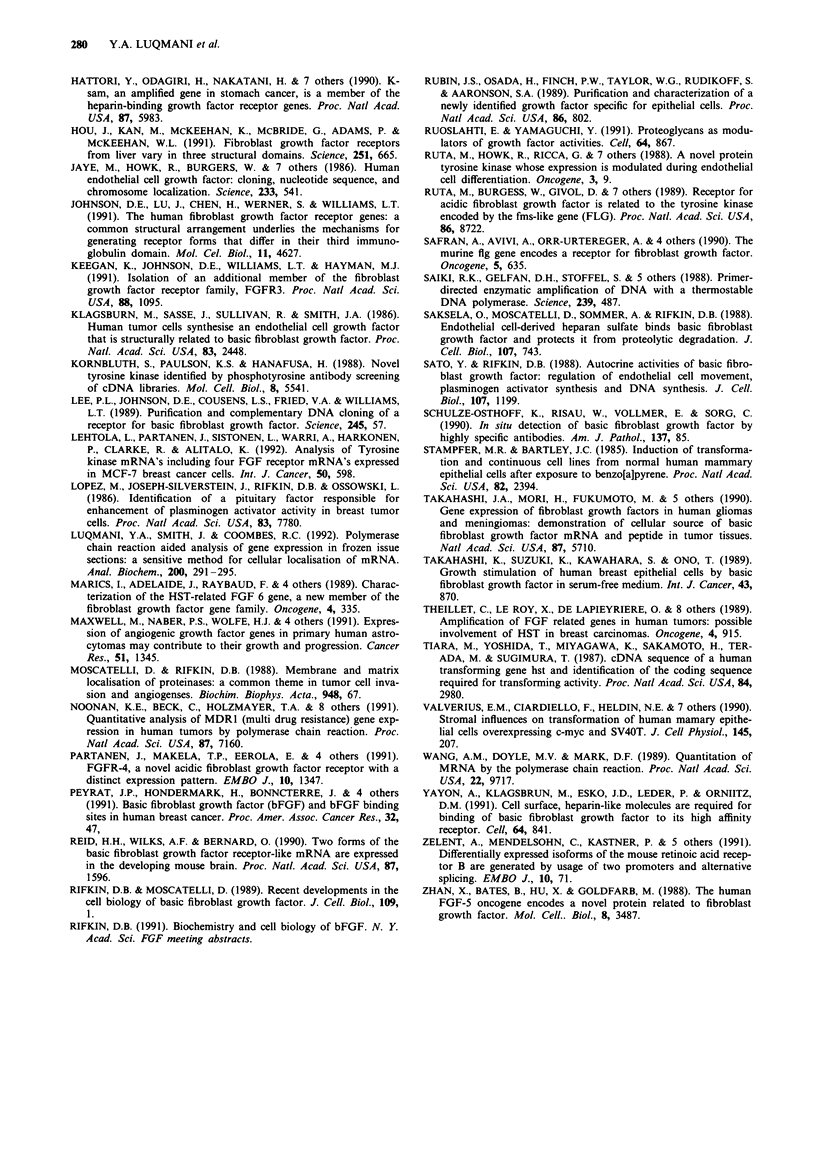# Expression of basic fibroblast growth factor, FGFR1 and FGFR2 in normal and malignant human breast, and comparison with other normal tissues.

**DOI:** 10.1038/bjc.1992.256

**Published:** 1992-08

**Authors:** Y. A. Luqmani, M. Graham, R. C. Coombes

**Affiliations:** Department of Medical Oncology, Charing Cross Hospital Medical School, London, UK.

## Abstract

**Images:**


Br. J. Cancer (1992), 66, 273-280 © Macmillan Press Ltd., 1992

### Expression of basic fibroblast growth factor, FGFR1 and FGFR2 in normal and malignant human breast, and comparison with other normal tissues

Y.A. Luqmani, M. Graham & R.C. Coombes

*Department of Medical Oncology, Charing Cross Hospital Medical School, St Dunstan's Road, London W6 8RF, UK.*

**Summary** The expression of basic fibroblast growth factor (bFGF) and two of its receptors, FGFR1 and FGFR2, was detected using the polymerase chain reaction, and quantified by comparison to the relative amount of product obtained following co-amplification of the ubiquitous glyceraldehyde phosphate dehydrogenase transcript. Varying levels were found in the vast majority of both cancer and non-malignant breast biopsies as well as in samples of several other normal human tissues. Significantly less bFGF was present in cancers ($P < 0.0001$). Similarly, FGFR2 product was also much less in cancer tissues ($P = 0.0078$), as was FGFR1 ($P = 0.002$). FGFR1 levels in cancers tended to be higher in those which were oestrogen receptor positive ($P < 0.06$). Amplification of different coding regions showed evidence of variant forms of FGFR1 RNA. Cancers appeared to have a significantly greater proportion of PCR product corresponding to the region between the third immunoglobulin like domain and the tyrosine kinase domain ($P = 0.046$). Differential expression was observed in breast cell lines, with bFGF in the normal derived HBL100, HBR SV1.6.1 and 184A1 but little or none in ZR-75-1, MCF-7, T47D and MDA-MB-231. FGFR1 was present in most of these but FGFR2 was absent from T47D, MDA-MB-231 and HBL100. ZR-75-1 cells had a marked preponderance of FGFR1 variants lacking part of the coding sequence.

Aberrant receptor processing may provide clues concerning the role of FGF's and their potential involvement in malignancy.

The fibroblast growth factors (FGF), acidic FGF, basic FGF, int-2, hst/k-FGF, FGF 5, FGF 6 and keratinocyte growth factor (Klagsburn *et al.*, 1986; Gospodarowicz *et al.*, 1986; Dickson & Peters, 1987; Tiara *et al.*, 1987; Delli Bovi *et al.*, 1987; Zhan *et al.*, 1988; Rubin *et al.*, 1989; Marics *et al.*, 1989) are a group of structurally related heparin binding polypeptide mitogens of widespread tissue distribution that induce proliferation of most cultured cells derived from embryonic mesoderm and neuroectoderm, including endothelial cells. Sharing 33-55% amino acid sequence identity and similar genomic organisation, they are involved in differentiation, embryogenesis, angiogenesis, chemotaxis and wound healing (Baird *et al.*, 1987; Burgess & Maciag, 1989; Rifkin & Moscatelli, 1989; Maxwell *et al.*, 1991).

The hst and int-2 genes are co-amplified in a minority of human cancers, including breast (Theillet *et al.*, 1989) but are poorly expressed. Acidic FGF expression is largely confined to neural tissues (Maxwell *et al.*, 1991) whereas bFGF is ubiquitously distributed in normal human tissues (Cordon-Cardo *et al.*, 1990), and in neural and squamous carcinomas, melanomas, osteosarcomas and hemangiomas (Takahashi *et al.*, 1990; Shulze-Osthoff *et al.*, 1990). It is mitogenic for cultured mammary epithelial cells (Takahashi *et al.*, 1989; Briozzo *et al.*, 1991) and stimulates plasminogen activator (Lopez *et al.*, 1986) which has been implicated in tumour invasion (Moscatelli & Rifkin, 1988). Basic FGF has been immunolocalised in myoepithelial cells in benign breast and around intraduct carcinomas but was not detected in cancer cells (Gomm *et al.*, 1991).

The cellular response to these peptides is mediated through cell surface receptors composed of several extracellular immunoglobulin-like domains, a transmembrane region and an intracellular portion exhibiting intrinsic tyrosine kinase activity (Ruta *et al.*, 1988; 1989; Lee *et al.*, 1989; Safran *et al.*, 1990). Several distinct human genes named flg (FGFR1), bek (FGFR2), FGFR3 and K-sam (also FGFR2) bearing sequence homology to the chick FGF receptor gene (Lee *et al.*, 1989) have been described (Dionne *et al.*, 1990; Keegan *et al.*, 1991; Hattori *et al.*, 1990). A fourth gene termed FGFR4 has also been reported (Partanen *et al.*, 1991). For the FGFR1 (Fujita *et al.*, 1991) and probably also the FGFR2 (Dionne *et al.*, 1990), multiple forms of the receptor have been found comprising deletions within the extracellular domain, apparently generated by alternatively spliced mRNA's. Variant forms of the murine receptor appear to be developmentally regulated in neuroepithelium (Reid *et al.*, 1990).

In view of the diversity of action of the FGF peptides and the apparent complexity in the processing of receptor isoforms, there is considerable potential for their involvement in malignant transformation, as well as in normal cellular interactions. In this report we describe the results of a study designed to assess the expression of bFGF and of the FGFR1 and FGFR2 receptors in human breast tissues. We have compared the levels of these mRNA's in normal/benign and cancerous biopsies with cultured cell lines and a variety of normal human tissues. Our findings indicate widespread expression of bFGF, FGFR1 and FGFR2, and suggest that many of these tissues contain variant FGFR1 receptor mRNA's in differing ratios.

### Materials and methods

### *Chemicals*

$^{32}$P dCTP (3000 Ci mmol$^{-1}$) was obtained from Amersham (UK), random hexamers, pdN$_6$, and dNTP's were from Pharmacia (Uppsala), MMLV reverse transcriptase was from GIBCO BRL (Pailsey, UK) and Taq polymerase was from Penninsula Laboratories (UK). Tissue culture media and foetal calf serum were from GIBCO (Paisley, UK). All other reagents were obtained from Sigma (Dorset, UK) unless indicated and were of the highest available grade.

### *Tissue samples*

Normal, benign and malignant breast biopsies were obtained from patients attending the Breast Clinics at St George's, the Royal Marsden and allied hospitals in London. No treatment had been given prior to surgery. Following histological con-

Correspondence: Y.A. Luqmani.
Received 24 February 1992; and in revised form 10 April 1992.

firmation of diagnosis, samples were dissected for material of interest and snap frozen and stored in liquid nitrogen. Tissues were examined from 66 primary carcinomas from patients aged between 29 and 79 years. Details of these patients are given in Table I. The non-malignant samples were composed of tissue removed from adjacent to cancer or from benign tumour. Other tissues used in this study were either surgical biopsy specimens or autopsy material.

*Probes*

The following cDNA inserts were used in this study: bFGF, 0.475 Kb excised from PBR322 (Abraham *et al.*, 1986) FGFR1, 1.2 Kb excised from pGEM1 (Ruta *et al.*, 1988) and FGFR2 excised from pGEM1 (Dionne *et al.*, 1990). Hybridisations were initially performed using these clones, but after the identity of the PCR products had been independently verified (i.e. by restriction enzyme anaylsis), we used PCR products for labelling.

*Cell culture*

The following breast cell lines (Engel & Young, 1978) were maintained in continuous culture in Dulbecco's minimal essential medium containing 10% foetal calf serum: MCF-7 (Michigan Cancer Foundation USA), T47D (Dr H. Freake), ZR-75-1 (Dr M. Lippman) MDA-MB-231 (Mason Research Institute, Rockville, MD, USA), HBR SV1.6.1 (Dr M. O'Hare), and HBL100. Cell pellets of 184A1 and 184B5 breast lines (Stampfer & Bartley, 1985) were provided by Dr M. Stampfer. The gastric line, KATO 111, was from Dr T. Motoyama and was maintained in RPMI-1640 medium containing 10% foetal calf serum. Cell pellets from squamous carcinoma lines, SMN, GEE and PAP were obtained from Dr B. Gusterson. The rhabdomyosarcoma cell line, A204, was from Dr C. Cooper.

*RNA isolation*

Total cellular RNA was extracted from frozen tissue using the guanidinium isothiocyanate procedure (Chirgwin *et al.*, 1979), and a modified technique (Chomczynski & Saatchi, 1987) utilising RNAZOL (Biogenesis, Bournemouth, UK) was used for cell lines. For Northern analysis, glyoxal-denatured RNA was electrophoresed on 1% agarose, transferred to Hybond N membrane and hybridised with random primer labelled (Feinberg & Vogelstein, 1983) cDNA.

*Reverse transcription*

First strand cDNA was synthesised using MMLV reverse transcriptase. RNA (2 µg in 12 µl) was boiled, snap cooled and added to 1 µl of enzyme (200 units), 4 µl 5 × reaction buffer (250 mM Tris-HCl, pH 8.3, 375 mM KCl, 15 mM $MgCl_2$), 1 µl dNTP (20 mM each dATP, dCTP, dGTP and dTTP), 1 µl dithiothreitol and 1 µl random hexamers (250 ng). Following incubation at 37–42° for 1 h, the mixture was heated to 95°, snap cooled and stored at −20°C. With each experiment an additional tube which contained all the reagents except the enzyme was always included as a blank control.

*Polymerase chain reaction amplification*

Specific cDNA sequences were amplified (Saiki *et al.*, 1988) in a reaction mix (100 µl) composed of 1–4 µl cDNA (equivalent to 100–400 ng RNA), 2 units Taq polymerase, 200 µM dNTP, 200 ng each of the 5′ and 3′ sequence specific primers in various combinations and buffer containing (in final concentrations) 8 mM Tris-HCl pH 8.4, 40 mM KCl, 1.5 mM $MgCl_2$ and 0.02% Tween, and overlaid with mineral oil. In the standard procedure, 18 cycles of amplification were performed with denaturation for 30 s at 94°, annealing for 1 min at 45° and extension at 72° for 1 min with an extra 9 min extension for the last cycle. An aliquot (25 µl) was removed for measurement of GAP and the reaction continued for a further 12 or 22 cycles using the same parameters for the other gene products. All samples were analysed on at least two separate occasions to check for reproducibility. If there was more than 20% variation samples were re-done.

In preliminary experiments to establish the conditions under which the levels in individual samples could be compared, the input cDNA was varied from 3–200 ng, and cycle number from 4–40.

**Table I** Details of patients studied

| *Characteristic* | *No.* | *%* |
|---|---|---|
| Total no. | 66 | |
| Age range | 29–79 | |
| Mean age | 65 | |
| Menopausal status | | |
| pre | 15 | 31 |
| post | 33 | 69 |
| not known | 18 | |
| T stage | | |
| $T_0$ | 2 | 4 |
| $T_1$ | 15 | 33 |
| $T_2$ | 20 | 43 |
| $T_3$ | 6 | 13 |
| $T_4$ | 3 | 7 |
| not known | 20 | |
| Histological node status | | |
| negative | 30 | 68 |
| positive | 14 | 32 |
| not known | 22 | |
| ER status | | |
| positive | 17 | 61 |
| negative | 11 | 39 |
| not known | 38 | |
| Pathological size (mm) | | |
| 10–20 | 21 | 55 |
| >20 | 17 | 45 |
| not known | 28 | |
| Histological type | | |
| infiltrating ductal | 36 | 90 |
| infiltrating lobular | 4 | 10 |
| other | 4 | |
| not known | 22 | |

*Gel electrophoresis, blotting and hybridisation*

Aliquots of chloroform extracted PCR products (10 µl) were electrophoresed in 1.5% agarose in Tris acetate EDTA buffer pH 8 containing ethidium bromide. Care was taken to ensure that exact amounts were loaded, this being essential for quantitative comparisons.

For blotting, gels were soaked briefly in 0.4 M NaOH, and DNA transferred onto Hybond $N^+$ membrane in the same buffer. For hybridisation, filters were placed in roller bottles (Hybaid, UK) with a solution (60 µl $cm^{-2}$) containing 50% (v/v) formamide, 0.1% SDS, 5 × Denhardts (0.1% each of polyvinylpyrrolidine, bovine serum albumin and ficoll), 5 mM EDTA, 75 mM NaCl and 250 µg $ml^{-1}$ denatured sonicated salmon sperm DNA, and incubated at 42° for 4–6 h. After this time, the relevant probe (either cDNA or PCR product) labelled with $^{32}$P-dCTP (to specific activities between $5.10^8$ to $10^9$ c.p.m. $\mu g^{-1}$ DNA) using the random primer method (Feinberg & Vogelstein, 1983) was added and incubation continued for a further 16–20 h. Filters were subsequently washed in 2 × SSC, 0.1% SDS at 20°C for 25 min with five changes of buffer, and then at 60–65°C in 0.1 × SSC, 0.1% SDS for 1 h, with two changes of buffer, and exposed to Amersham hyperfilm at −70°C using intensifying screens for periods between 30 min to several days. Band intensities were quantified using a laser densitometer.

*Calculation of results*

To compare the levels of expression of the bFGF, FGFR1 and FGFR2 genes in different samples, the densitometric readings were expressed as a ratio of the signal obtained for

GAP, a ubiquitously expressed gene encoding a common glycolytic enzyme. These values were normalised against the value obtained for A204 (for bFGF and FGFR1) or KATO 111 (for FGFR2) cells, a sample of which was included on every run and was present on each blot. The final value was calculated as below:

$$\frac{\text{Signal for FGFR (sample).}}{\text{signal for GAP (sample).}} \times \frac{\text{signal for FGFR (A204).*}}{\text{signal for GAP (A204).}}$$

* or KATO 111 as appropriate.

*Statistical tests*

In order to compare the two unequal groups of cancer and non-malignant tissues, both with skewed distributions, we used a non-parametric test, the Mann Whitney U test, which gave a two tailed probability. The data was also analysed by the Kruskal-Wallis one-way analysis of variance by ranks and gave the same probability values.

*Restriction enzyme analysis*

To verify identity of PCR products, a sample was ethanol precipitated following a phenol extraction, resuspended in water and digested for 3 h with the appropriate restriction enzyme. An aliquot was electrophoresed on 1% agarose and bands visualised with ethidium bromide.

*Oligonucleotide primers*

Oligonucleotides were synthesised on a Cyclone Plus DNA Synthesiser (Milligan Bioresearch, Massachusetts, USA) by phosphoramidite chemistry, deprotected by treatment with $NH_4OH$ for 5-6 h at 55°C, vacuum dried, resuspended in water and used without further purification.

The primer pairs (Table II) were designed such that the intervening sequence contained at least one intron. The functional domains of the FGFR's encoding the regions amplified are shown in Figure 1.

### Results

We were unable to detect the presence of mRNA for either FGFR1 or FGFR2 using conventional Northern blotting methods, with up to 20 µg of total RNA extracted from breast tissues, under conditions in which the appropriate bands could be seen with A204 or KATO 111 cells. Use of poly $A^+$ RNA could increase sensitivity, but tumour tissues generally yielded too little RNA for poly $A^+$ selection. We therefore used the PCR technique to perform this study.

*Optimisation of PCR conditions*

Figure 2 (inset) shows the accumulation of GAP PCR product with varying number of amplification cycles, using an input of 100 ng of RT product from the RNA of a tumour sample taken at random. Whilst ethidium bromide stained bands were visible after 20 cycles, product was detected after 12 cycles by hybridisation with $^{32}P$-labelled GAP cDNA, and increased linearly between 10-30 cycles. The mid-range, 18 cycles, was therefore selected for measuring GAP levels. Product accumulation was directly proportional to input template (3-200 ng RNA) after 18 cycles (Figure 2). The reproducibility of the method was assessed in several experiments in which the RT product from 2 µg RNA from 13

**Figure 1** Schematic representation of the functional domains of the FGFR to show the approximate areas included in the RNA coding sequence amplified by the FGFR primers used in this study.

**Figure 2** GAP PCR products, accumulated from 3-200 ng RT product from a random breast tumour sample after 18 cycles of amplification, were electrophoresed in 1.5% agarose, blotted onto Hybond N+ membrane and hybridised with $^{32}P$-labelled GAP cDNA. Autoradiographic signals obtained following exposure to Hyperfilm were quantified using laser densitometry. Similar results were obtained with two other samples. Inset shows accumulation of GAP PCR product with increasing number of amplification cycles using 100 ng RT product synthesised from the RNA of a randomly selected breast tumour sample. The arrow indicates the approximate mid range of the linear part of the curve. These results were reproduced using at least four other samples, on separate occasions.

**Table II** Oligonucleotide primers

| *RNA* | *Primer sequence* | *Product size (bp)* | *Reference* |
|---|---|---|---|
| bFGF | CTGTACTGCAAAAACGGG<br>AAAGTATAGCTTTCTGCC | 349 | Abraham *et al.*, 1986 |
| FGFR1 (A) | CCTCTTCTGGGCTGTGCT<br>CGGGCATACGGTTTGGTT | 433 | Dionne *et al.*, 1991 |
| FGFR1 (B) | GACAAAGAGATGGAGGTGCT<br>GTTGTAGCAGTATTCCAGCC | 801 | Dionne *et al.*, 1991 |
| FGFR2 | GGTCGTTTCATCTGCCTGGT<br>CCTTCCCGTTTTTCAGCCAC | 578 | Dionne *et al.*, 1991 |
| GAP | TCCCATCACCATCTTCCA<br>CATCACGCCACAGTTTCC | 379 | Arcari *et al.*, 1984 |

**Figure 3** Southern blot showing GAP PCR products. To evaluate the reproducibility of the PCR technique, RT products (equivalent of 100 ng), from 2 μg of 13 breast tumour RNA samples, were amplified through 18 cycles of PCR using GAP primers alone **a**, or in the presence of either FGFR1 B primers **b**, or of FGFR1 A and FGFR2 primers **c**. Aliquots were electrophoresed through 1.5% agarose, blotted only Hybond N+ membrane and hybridised with $^{32}$P-labelled GAP cDNA. Samples from **a** and **b** were run on the same gel and all three were hybridised and exposed to Hyperfilm together to achieve identical conditions. Control samples run in lanes marked with an asterisk, were from PCR reactions using RNA from mock RT reactions in which the reverse transcriptase had been ommitted.

samples was divided into three portions and GAP amplified (a), alone (b), in the presence of FGFR1 primers B or (c), in the presence of FGFR1 A and FGFR2 primers (Figure 4): the variation was generally no greater than 50%, and the yield of GAP product was not greatly affected by the presence of the other primers. The yield of product from the FGFR1 and FGFR2 primers was reduced when GAP primers were present; but independently of template. The bFGF primers would only work on their own. Thus the optimal primer combinations used were (a), GAP and FGFR1 B (b), GAP and FGFR1 A and FGFR2 and (c), bFGF alone. In the latter case it was necessary to amplify the GAP control separately, but to maximise uniformity, complete double volume PCR mixes containing cDNA were divided into two tubes containing the respective primers, and amplification performed in adjacent wells of the Thermocycler. Forty cycles of PCR were optimal for detection of FGFR1 and FGFR2 and 30 cycles were most suitable for bFGF (Figure 4), being within the *linear* stage of amplification. The slope of the linear part of the curve for each primer set was very similar, suggesting similar amplification efficiencies under the particular conditions we used. It should also be noted that this linear amplification

**Figure 4** Accumulation of PCR products using bFGF (●), FGFR1 A (□), FGFR1 B (O) and FGFR2 (■) primers, in the combinations described in Methods, with aliquots (100 ng) of RT product from random tumour samples amplifed through 55 cycles. Equal aliquots, removed after indicated number of cycles, were electrophoresed through 1.5% agarose, blotted onto Hybond N+ membrane and hybridised with the appropriate $^{32}$P-labelled probe. Exposure to Hyperfilm was carried out at −70°C and autoradiographic signals quantified by laser densitometry. Data are from one of two such experiments showing similar results.

was occurring at a time when GAP amplification had plateaued after 30 cycles, in the same reaction mixture. Thus it is valid to sample different PCR products at different cycle numbers.

For all the genes concerned we verified the identity of the product both by demonstrating hybridisation to the appropriate cDNA and by site specific cleavage with restriction enzymes (data not shown).

### *Expression of bFGF in breast tissues*

All 66 biopsies taken from patients .with primary breast cancer, and 28 samples of either histologically normal tissue taken from adjacent to cancer or from patients with benign disease produced the expected 349 bp bFGF PCR product but the relative amounts varied greatly within both groups (Figure 5). The mean ± s.e.m. for the cancers was 0.87 ± 0.12 as compared with 4.25 ± 1.03 for the non-malignant group; these being significantly different ($P<0.0001$).

### *Expression of FGFR2 in breast tissues*

As with bFGF the individual levels of FGFR2 PCR product varied considerably in both cancers and non-malignant tissues alike (Figure 5). Apart from the expected 578 bp fragment, hybridisation to two smaller bands was also observed; the origin of these is being investigated in the light of data which has appeared since this work was completed (Hou *et al*., 1991). Only in three samples of cancer (<5%) was FGFR2 undetectable. Otherwise, there was a significantly greater level of amplified FGFR mRNA product in the normal tissues: means ± s.e.m. being 3.42 ± 0.46 and 9.92 ± 2.5 for cancers ($n = 66$) and non-malignant ($n = 31$) samples respectively (two-tailed probability = 0.0078).

### *Expression of FGFR1 in breast tissues*

We used two sets of primers to amplify either the region encoding the outermost immunoglobulin-like domain, or that spanning the sequence from the third immunoglobulin-like domain to, and including, half of the tyrosine kinase domain. Again a wide range of values were recorded for both cancers and non-malignant biopsies, with both pairs of primers (Figure 5). With FGFR1 B primers the two groups had means ± s.e.m. respectively of 0.97 ± 0.11 ($n = 66$) and 1.78 ± 0.39 which were not significantly different. Product was obtained with all samples. With FGFR1 A primers the means ± s.e.m. were 3.96 ± 0.62 ($n = 66$) and 12.6 ± 2.6 ($n = 31$) for cancers and non-malignant groups respectively.

**Figure 5** Scattergrams showing the relative amounts of PCR product accumulated after amplification of RT product (calculated as described in Methods) from cancer or non-malignant breast biospies. For bFGF the mean values of 0.87±0.12 (s.e.m.) and 4.25±1.03 respectively, were significantly different ($P<0.0001$). For FGFR2, the two groups had mean values of 3.42±0.46 (s.e.m.) and 9.92±2.5 respectively, which were significantly different ($P=0.0078$). For FGFR1 there was no difference between the two groups (means of 0.97±0.11 (s.e.m.) and 1.78±0.39 respectively) when primers B were used. With primers A, the mean values of the two groups (3.96±0.62 (s.e.m.) and 12.6±2.6 respectively) were significantly different ($P=0.0002$).

These two values were significantly different (two-tailed probability = 0.0002). In the cancers, two samples (<3%) had no detectable FGFR1 A product and three in the non-malignant group (10%). The relative amount of FGFR1 product varied considerably depending upon which region was being amplified. Thus, instead of the expected constant ratio for FGFR1 A: FGFR1 B product for all samples, we obtained widely different ratios. The mean values of the cancer and non-malignant groups were significantly different (means ± s.e.m. were 8.7 ± 1.5 ($n=63$) and 46.9 ± 25 ($n=27$) (two-tailed probability = 0.046).

*Relationship of bFGF, FGFR1 and FGFR2 expression to clinical parameters*

For those patients for whom clinical data was available, we analysed the results with respect to the major clinical features; T stage, nodal involvement and oestrogen receptor status. Table III shows the breakdown of this analysis, which was done by using a purely arbitary cut off point to divide the tumour samples into two groups, with (comparatively) high and low levels of expression. The numbers are still too small to permit accurate correlate into clinical parameters, but oestrogen receptor positivity appeared to be associated with high expression of the FGFR2 mRNA. However, due to the small numbers, this relationship was only marginally significant ($P<0.06$, Fishers exact two-sided probability).

*Expression of bFGF, FGFR1 and FGFR2 in cell lines*

We examined RNA from four cell lines derived from breast cancer (ZR-75, T47D, MCF-7 and MDA-MB-231), four derived from normal breast cells (HBL100, HBRSV1.6.1, 184A1 and 184B5) and three from squamous carcinomas (GEE, PAP and SMN) (Figure 6). There was no bFGF product in three of the breast cancer lines, and only a small amount in ZR-75-1 cells. In contrast, all four of the 'normal-derived' breast lines had levels comparable with the low-range values found in cancer tissues. Of the three squamous lines two gave detectable bFGF product.

The FGFR2 product was present in MCF-7 and in ZR-75-1 cells but undetectable in T47D and HBL100 cells. The highest levels were seen in HBR SV1.6.1 and the 184B5 cells. Only PAP of the squamous lines produced FGFR2. Using FGFR1 A primers, highest levels were found in ZR-75 (in the same range as cancer tissues) with lower levels in HBL100, HBRSV1.6.1 and T47D. Product was barely detectable in MCF-7, and absent in 184B5 and 184A1. In the squamous lines expression was observed in GEE and SMN but not PAP cells. With the FGFR1 B primers, the pattern was similar except that there were very low amounts for ZR-75-1.

*Expression of bFGF, FGFR1 and FGFR2 in a panel of normal human tissues*

Basic FGF was present in heart, ileum, colon, kidney, stomach, adrenal gland, ovary, skin and thyroid with less than 5 fold differences between them, except for lung, which was much higher. The FGFR2 gene was also ubiquitously expressed, except in heart. The highest amount was present in the thyroid specimen.

All samples expressed FGFR1 with highest levels in skin ovary, and heart (Figure 7).

**Table III** Relationship between bFGF, FGFR1 and FGFR2 PCR product levels and clinical status

| *RNA* | *Relative level of PCR prod.* | *Total no. patients* | *Stage* $T_0/T_2$ | *Stage* $T_3/T_4$ | *Nodes* − | *Nodes* + | *ER* − | *ER* + |
|---|---|---|---|---|---|---|---|---|
| bFGF | <0.6 | 36 | 26 | 3 | 20 | 10 | 8 | 11 |
| | >0.6 | 29 | 16 | 6 | 16 | 7 | 4 | 8 |
| FGFR1 | <3 | 39 | 24 | 4 | 19 | 11 | 9 | 8 |
| (A) | >3 | 27 | 19 | 5 | 18 | 6 | 3 | 10 |
| FGFR1 | <1 | 39 | 26 | 5 | 22 | 10 | 7 | 9 |
| (B) | >1 | 27 | 17 | 4 | 16 | 7 | 5 | 10 |
| FGFR2 | <2 | 38 | 26 | 5 | 23 | 9 | 10 | 8 |
| | >2 | 28 | 17 | 4 | 14 | 8 | 2 | 11 |

A and B refer to the primers used. Note that details not available for all patients.

**Figure 6** Histograms showing the accumulation of PCR products from RT products of cell line RNA, for bFGF, FGFR1 (primers A), FGFR1 (primers B) and FGFR2 as indicated. Experimental details are described in Methods.

**Figure 7** Histograms showing the accumulation of PCR products from RT products of RNA extracted from various normal human tissue samples, with bFGF, FGFR1 (primers A and B) and FGFR2 primers, as indicated. Experimental details as described in Methods.

### Discussion

This study describes the expression of bFGF and two of the four recently identified FGF receptors, FGFR1 and FGFR2 in a series of human breast biopsies, cell lines and in a panel of normal tissues. Due to their low abundance, we devised a sensitive PCR based protocol which relies on co-amplification of an internal ubiquitously expressed sequence, and reference to values determined for an arbitary standard sample. An advantage of using GAP, is that the level of this gene is likely to reflect the metabolic state of the cells and therefore its fluctuations would correct for differences due simply to differences in the proliferative activity, rather than specifically malignancy associated events. This procedure, similar to one described by Noonan *et al.* (1990) and unlike methods in which exogenously added cRNA is used (e.g. Wang *et al.* (1989)) overcomes the problems of variations in RNA quality and purity, sampling errors, and other differences in electrophoresis, gel blotting, hybridisation and autoradiographic exposure times. The main limitation is that our values are purely arbitary and the actual levels of the three genes cannot be compared with each other. Although bFGF had to be amplified separately from GAP, we ensured that buffer conditions were identical and both tubes had exactly the same amount of cDNA. Duplicates done in this way showed no more than 20% variation.

In apparent contrast with earlier immunohistochemical observations (Gomm *et al.*, 1991), we found that bFGF mRNA was present in breast cancer as well as benign tissue, though at significantly reduced levels. It is unclear whether this reflects the difference in sensitivity of the two methods, or differential translational control. All the breast cell lines derived from normal epithelia expressed the gene whilst only one of the four cancer derived lines did so and to a much lesser extent (more than 50 fold less). Valverius *et al.* (1990) reported the presence of bFGF mRNA in primary cultures of human mammary fibroblasts, but not in the transformed 184A1N4 epithelial cells. This discrepancy is probably due to the greater sensitivity of PCR over Northern blotting methods. We are currently doing cellular localisation using a combination of PCR with dissection of frozen tissue sections (Luqmani *et al.*, 1992) to determine the precise cellular origin of the amplified RNA. The widespread presence of bFGF (Cordon-Cardo *et al.* 1990) suggests an important role in normal cellular function, but the lack of a signal peptide (Jaye *et al.*, 1986), precluding secretion in the classically recognised manner, has posed problems regarding its potential as a locally produced para/autocrine factor. However its appearance in conditioned media (Sato & Rifkin, 1988; Rifkin, 1991) and its association with the extracellular matrix (Saksela *et al.*, 1988; Baird & Walicke, 1989) suggest that it may be externalised in conjuction with glycosaminoglycans which are also thought to modulate its biological activity (Ruoslahti & Yamaguchi, 1991; Yayon *et al.*, 1991).

Both FGFR1 and FGFR2 were expressed in all tissues except heart (an observation also made by Kornbluth *et al.* (1988) and Reid *et al.* (1990)) and in the vast majority of the breast tissues. The wide range of values reflects the sensitivity of the PCR. In the light of growing evidence for the existence of truncated FGFR isoforms generated by alternative splicing, affecting all three structural domains, our results with the FGFR1 primers suggest that these commonly co-exist in the same tissue but in greatly varying proportions. Thus the ratio of PCR products obtained using the two FGFR1 primer sets reflects a greater frequency of FGFR mRNA's with deletions in the outermost Ig like domains in the cancer tissues as compared with the non-malignant breast samples but that the truncated form is present in both groups. Fujita *et al.* (1991) observed this to be the major form in placenta and found that it still mediated biologic response to both acidic and bFGF, suggesting that this domain is not required for binding but may play some other facilitatory role. Another placental cDNA encoding a soluble form of the placental receptor contains only the first two Ig like domains has been expressed in CHO cells and shown to undergo oligomerisation and retain binding activity (Duan *et al.*, 1991). A truncated FGFR2 cDNA encoding only the signal peptide and the first Ig domain followed by a termination codon, and an FGFR1 clone with only the two outer Ig like regions have also been reported (Dionne *et al.*, 1990). Spliced variants for the K-SAM (FGFR2) receptor have also been described in gastric carcinoma cells (Hattori *et al.*, 1990). We found not only differential expression of FGFR2 and FGFR1 in the various cell lines studies, but also evidence of forms of the FGFR1 with deletions. ZR-75-1 cells appeared to have mRNA encoding only the external part. Hou *et al.* (1991) postulate the existence of up to 12 different variants of the FGFR1 receptor, which by extrapolation to the other three identified FGF receptors could extend this family to 48 isoforms. Johnson *et al.* (1991) have also recently described a number of different variants of FGFR1. The antisense primer of the FGFR1 A pair used in this study covers a region in which another variant involving a two amino acid deletion has been found: thus, lack of product with this primer may be due to such a variant in our samples, though it would have to be a predominant form. In the light of these recent findings, we are currently designing new primers to map these regions.

Other receptors also appear to display variant types. For example, developmentally regulated isoforms of the murine retinoic acid receptor beta generated by alternative splicing and differential promoter usage have also been described and may reflect functional specificities (Zelent *et al.*, 1991).

The presence of receptors on several breast cell lines has been demonstrated by ligand binding studies (Peyrat *et al.*, 1991; Briozzo *et al.*, 1991) and by Northern analysis, using 6 μg mRNA, in MCF7 cells (Lehtola *et al.*, 1992). Mitogenic effects have been noted on both MCF-7 cells and on immortalised mammary epithelial cells derived from breast reduction mammoplaasty (Valverius *et al.*, 1990). Primary monolayer cultures of breast epithelial cells grown out of fragmented biopsy tissue were reported to show a modest response to bFGF (more pronounced in cancer than in non-malignant samples) but were unaffected by aFGF (Takahashi *et al.*, 1989). As none of these studies were concerned with the identity of the receptor, it is not yet clear which of the sub-types are involved. Our results suggest that FGFR1 mediated events could be studied using T47D, MDA-MB-231 and HBL100 lines, all of which appear to be FGFR2 negative. The FGFR1 negative squamous line, PAP, could be used to study FGFR2 action. This of course does not take into account the presence of FGFR3 and FGFR4, the expression of which has yet to be determined.

A recent survey (Adnane *et al.*, 1991) of 387 breast carcinomas showed amplification of both the FGFR1 and FGFR2 genes in about 12% of cases: FGFR1 amplification was correlated with nodal metastases and amplification of the HST, Int 2 and BCL 1 genes, and FGFR2 with c-myc.

This work was supported by the Cancer Research Campaign. We thank Caroline Mortimer for help with the clinical data.

### References

ABRAHAM, J.A., WHANG, J.L., TUMOLO, A. & 4 others (1986). Human basic fibroblast growth factor: nucleotide sequence and genomic organization. *EMBO J.*, **5**, 2523.

ADNANE, J., GAUDRAY, P., DIONNE, C.A. & 6 others (1991). BEK and FLG, two receptors to members of the FGF family, are amplified in subsets of human breast cancers. *Oncogene*, **6**, 659.

ARCARI, P., MARTINELLI, R. & SALVATORE, F. (1984). The complete sequence of a full length cDNA for human liver glyceraldehyde-3-phosphate dehydrogenase: evidence for multiple mRNA species. *Nucleic Acids Res.*, **12**, 9179.

BAIRD, A., UENO, N., ESCH, F. & LING, N. (1987). Distribution of fibroblast growth factors (FGF's) in tissues and structure-function studies with synthetic fragments of basic FGF. *J. Cell Physiol.*, **5** (Suppl) 101.

BAIRD, A. & WALICKE, P.A. (1989). Fibroblast growth factors. *Br. Med. Bull.*, **45**, 438.

BRIOZZO, P., BADET, J., CAPONY, F. & 4 others (1991). MCF7 mammary cancer cells respond to bFGF and internalize it following its release from extracellular matrix: a permissive role of cathepsin D. *Exp. Cell Res.*, **194**, 252.

BURGESS, W.H. & MACIAG, T. (1989). The heparin-binding (fibroblast) growth factor family of proteins. *Annu. Rev. Biochem.*, **58**, 575.

CHIRGWIN, S.M., PRZYBYLA, A.E., MACDONALD, R.J. & RUTTER, W.J. (1979). Isolation of biologically active ribonucleic acid from sources enriched in ribonucleases. *Biochemistry*, **18**, 5294.

CHOMCZYNSKY, P. & SAATCHI, N. (1987). Single-step method of RNA isolated by acid guanidinium thiocyanate-phenol-chloroform extraction. *Biochemistry*, **162**, 156–159.

CORDON-CARDO, C., VLODAVSKY, I., HAIMOVITZ-FRIEDMAN, A., HICKLIN, D. & FUKS, Z. (1990). Expression of basic fibroblast growth factor in normal human tissues. *Lab. Invest.*, **63**, 832.

DELLI BOVI, P., CURATOLA, A.M., KERN, F.G., GRECO, A., ITTMANN, M. & BASILICO, C. (1987). An oncogene isolated by transfection of Kaposi's sarcoma DNA encodes a growth factor that is a member of the FGF family. *Cell*, **50**, 729.

DICKSON, C. & PETERS, G. (1987). Potential oncogene product related to growth factors. *Nature*, **326**, 833.

DIONNE, C.A., CRUMLEY, G., BELLOT, F. & 6 others (1990). Cloning and expression of two distinct high affinity receptors cross-reacting with acidic and basic fibroblast growth factors. *EMBO J.*, **9**, 2685.

DUAN, D.R., WERNER, S., LEE, P. & WILLIAMS, L.T. (1991). Expression and characterisation of a soluble fibroblast growth factor receptor. *N Y Acad. Sci. FGF Meeting Abstract*, 41.

ENGEL, L.W. & YOUNG, N.A. (1978). Human breast carcinoma cells in continuous culture: a review. *Cancer Res.*, **38**, 4327.

FEINBERG, A.P. & VOGELSTEIN, B. (1983). A technique for radiolabelling DNA restriction endonuclease fragments to high specific activity. *Anal. Biochem.*, **132**, 6.

FUJITA, H., OHTA, M., KAWASAKI, T. & ITOH, N. (1991). The expression of two isoforms of the human fibroblast growth factor receptor (flg) is directed by alternative splicing. *Biochem. Biophys. Res. Comm.*, **174**, 946.

GOMM, J.J., SMITH, J., RYALL, G.K., BAILLIE, R., TURNBULL, L. & COOMBES, R.C. (1991). Localisation of fibroblast growth factor and transforming growth factor b1 in the human mammary gland. *Cancer Res.*, **51**, 4685.

GOSPODAROWICZ, D., NEUFELD, D. & SCHWEIGERER, L. (1986). Molecular and biological characterisation of fibroblast growth factor, an angiogenic factor which controls the proliferation and differentiation of mesoderm and neuroectoderm derived cells. *Cell. Diff.*, **19**, 1.

HATTORI, Y., ODAGIRI, H., NAKATANI, H. & 7 others (1990). K-sam, an amplified gene in stomach cancer, is a member of the heparin-binding growth factor receptor genes. *Proc. Natl Acad. USA*, **87**, 5983.

HOU, J., KAN, M., MCKEEHAN, K., MCBRIDE, G., ADAMS, P. & MCKEEHAN, W.L. (1991). Fibroblast growth factor receptors from liver vary in three structural domains. *Science*, **251**, 665.

JAYE, M., HOWK, R., BURGERS, W. & 7 others (1986). Human endothelial cell growth factor: cloning, nucleotide sequence, and chromosome localization. *Science*, **233**, 541.

JOHNSON, D.E., LU, J., CHEN, H., WERNER, S. & WILLIAMS, L.T. (1991). The human fibroblast growth factor receptor genes: a common structural arrangement underlies the mechanisms for generating receptor forms that differ in their third immunoglobulin domain. *Mol. Cel. Biol.*, **11**, 4627.

KEEGAN, K., JOHNSON, D.E., WILLIAMS, L.T. & HAYMAN, M.J. (1991). Isolation of an additional member of the fibroblast growth factor receptor family, FGFR3. *Proc. Natl Acad. Sci. USA*, **88**, 1095.

KLAGSBURN, M., SASSE, J., SULLIVAN, R. & SMITH, J.A. (1986). Human tumor cells synthesise an endothelial cell growth factor that is structurally related to basic fibroblast growth factor. *Proc. Natl. Acad. Sci. USA*, **83**, 2448.

KORNBLUTH, S., PAULSON, K.S. & HANAFUSA, H. (1988). Novel tyrosine kinase identified by phosphotyrosine antibody screening of cDNA libraries. *Mol. Cell. Biol.*, **8**, 5541.

LEE, P.L., JOHNSON, D.E., COUSENS, L.S., FRIED, V.A. & WILLIAMS, L.T. (1989). Purification and complementary DNA cloning of a receptor for basic fibroblast growth factor. *Science*, **245**, 57.

LEHTOLA, L., PARTANEN, J., SISTONEN, L., WARRI, A., HARKONEN, P., CLARKE, R. & ALITALO, K. (1992). Analysis of Tyrosine kinase mRNA's including four FGF receptor mRNA's expressed in MCF-7 breast cancer cells. *Int. J. Cancer*, **50**, 598.

LOPEZ, M., JOSEPH-SILVERSTEIN, J., RIFKIN, D.B. & OSSOWSKI, L. (1986). Identification of a pituitary factor responsible for enhancement of plasminogen activator activity in breast tumor cells. *Proc. Natl Acad. Sci. USA*, **83**, 7780.

LUQMANI, Y.A., SMITH, J. & COOMBES, R.C. (1992). Polymerase chain reaction aided analysis of gene expression in frozen issue sections: a sensitive method for cellular localisation of mRNA. *Anal. Biochem.*, **200**, 291–295.

MARICS, I., ADELAIDE, J., RAYBAUD, F. & 4 others (1989). Characterization of the HST-related FGF 6 gene, a new member of the fibroblast growth factor gene family. *Oncogene*, **4**, 335.

MAXWELL, M., NABER, P.S., WOLFE, H.J. & 4 others (1991). Expression of angiogenic growth factor genes in primary human astrocytomas may contribute to their growth and progression. *Cancer Res.*, **51**, 1345.

MOSCATELLI, D. & RIFKIN, D.B. (1988). Membrane and matrix localisation of proteinases: a common theme in tumor cell invasion and angiogenses. *Biochim. Biophys. Acta.*, **948**, 67.

NOONAN, K.E., BECK, C., HOLZMAYER, T.A. & 8 others (1991). Quantitative analysis of MDR1 (multi drug resistance) gene expression in human tumors by polymerase chain reaction. *Proc. Natl Acad. Sci. USA*, **87**, 7160.

PARTANEN, J., MAKELA, T.P., EEROLA, E. & 4 others (1991). FGFR-4, a novel acidic fibroblast growth factor receptor with a distinct expression pattern. *EMBO J.*, **10**, 1347.

PEYRAT, J.P., HONDERMARK, H., BONNCTERRE, J. & 4 others (1991). Basic fibroblast growth factor (bFGF) and bFGF binding sites in human breast cancer. *Proc. Amer. Assoc. Cancer Res.*, **32**, 47,

REID, H.H., WILKS, A.F. & BERNARD, O. (1990). Two forms of the basic fibroblast growth factor receptor-like mRNA are expressed in the developing mouse brain. *Proc. Natl. Acad. Sci. USA*, **87**, 1596.

RIFKIN, D.B. & MOSCATELLI, D. (1989). Recent developments in the cell biology of basic fibroblast growth factor. *J. Cell. Biol.*, **109**, 1.

RIFKIN, D.B. (1991). Biochemistry and cell biology of bFGF. *N. Y. Acad. Sci. FGF meeting abstracts.*

RUBIN, J.S., OSADA, H., FINCH, P.W., TAYLOR, W.G., RUDIKOFF, S. & AARONSON, S.A. (1989). Purification and characterization of a newly identified growth factor specific for epithelial cells. *Proc. Natl Acad. Sci. USA*, **86**, 802.

RUOSLAHTI, E. & YAMAGUCHI, Y. (1991). Proteoglycans as modulators of growth factor activities. *Cell*, **64**, 867.

RUTA, M., HOWK, R., RICCA, G. & 7 others (1988). A novel protein tyrosine kinase whose expression is modulated during endothelial cell differentiation. *Oncogene*, **3**, 9.

RUTA, M., BURGESS, W., GIVOL, D. & 7 others (1989). Receptor for acidic fibroblast growth factor is related to the tyrosine kinase encoded by the fms-like gene (FLG). *Proc. Natl. Acad. Sci. USA*, **86**, 8722.

SAFRAN, A., AVIVI, A., ORR-URTEREGER, A. & 4 others (1990). The murine flg gene encodes a receptor for fibroblast growth factor. *Oncogene*, **5**, 635.

SAIKI, R.K., GELFAN, D.H., STOFFEL, S. & 5 others (1988). Primer-directed enzymatic amplification of DNA with a thermostable DNA polymerase. *Science*, **239**, 487.

SAKSELA, O., MOSCATELLI, D., SOMMER, A. & RIFKIN, D.B. (1988). Endothelial cell-derived heparan sulfate binds basic fibroblast growth factor and protects it from proteolytic degradation. *J. Cell. Biol.*, **107**, 743.

SATO, Y. & RIFKIN, D.B. (1988). Autocrine activities of basic fibroblast growth factor: regulation of endothelial cell movement, plasminogen activator synthesis and DNA synthesis. *J. Cell. Biol.*, **107**, 1199.

SCHULZE-OSTHOFF, K., RISAU, W., VOLLMER, E. & SORG, C. (1990). *In situ* detection of basic fibroblast growth factor by highly specific antibodies. *Am. J. Pathol.*, **137**, 85.

STAMPFER, M.R. & BARTLEY, J.C. (1985). Induction of transformation and continuous cell lines from normal human mammary epithelial cells after exposure to benzo[a]pyrene. *Proc. Natl Acad. Sci. USA*, **82**, 2394.

TAKAHASHI, J.A., MORI, H., FUKUMOTO, M. & 5 others (1990). Gene expression of fibroblast growth factors in human gliomas and meningiomas: demonstration of cellular source of basic fibroblast growth factor mRNA and peptide in tumor tissues. *Natl Acad. Sci. USA*, **87**, 5710.

TAKAHASHI, K., SUZUKI, K., KAWAHARA, S. & ONO, T. (1989). Growth stimulation of human breast epithelial cells by basic fibroblast growth factor in serum-free medium. *Int. J. Cancer*, **43**, 870.

THEILLET, C., LE ROY, X., DE LAPIEYRIERE, O. & 8 others (1989). Amplification of FGF related genes in human tumors: possible involvement of HST in breast carcinomas. *Oncogene*, **4**, 915.

TIARA, M., YOSHIDA, T., MIYAGAWA, K., SAKAMOTO, H., TERADA, M. & SUGIMURA, T. (1987). cDNA sequence of a human transforming gene hst and identification of the coding sequence required for transforming activity. *Proc. Natl Acad. Sci. USA*, **84**, 2980.

VALVERIUS, E.M., CIARDIELLO, F., HELDIN, N.E. & 7 others (1990). Stromal influences on transformation of human mamary epithelial cells overexpressing c-myc and SV40T. *J. Cell Physiol.*, **145**, 207.

WANG, A.M., DOYLE, M.V. & MARK, D.F. (1989). Quantitation of MRNA by the polymerase chain reaction. *Proc. Natl Acad. Sci. USA*, **22**, 9717.

YAYON, A., KLAGSBRUN, M., ESKO, J.D., LEDER, P. & ORNIITZ, D.M. (1991). Cell surface, heparin-like molecules are required for binding of basic fibroblast growth factor to its high affinity receptor. *Cell*, **64**, 841.

ZELENT, A., MENDELSOHN, C., KASTNER, P. & 5 others (1991). Differentially expressed isoforms of the mouse retinoic acid receptor B are generated by usage of two promoters and alternative splicing. *EMBO J.*, **10**, 71.

ZHAN, X., BATES, B., HU, X. & GOLDFARB, M. (1988). The human FGF-5 oncogene encodes a novel protein related to fibroblast growth factor. *Mol. Cell.. Biol.*, **8**, 3487.